# Supplementary material for: Programmed cell death regulator BAP2 is required for IRE1-mediated unfolded protein response in Arabidopsis
Source: Nat Commun. 2024 Jul 10;15:5804. doi: 10.1038/s41467-024-50105-6 (PMC11237027; doi:10.1038/s41467-024-50105-6)
Supplement: Supplementary file 1 — Supplementary Information [file 41467_2024_50105_MOESM1_ESM.docx]

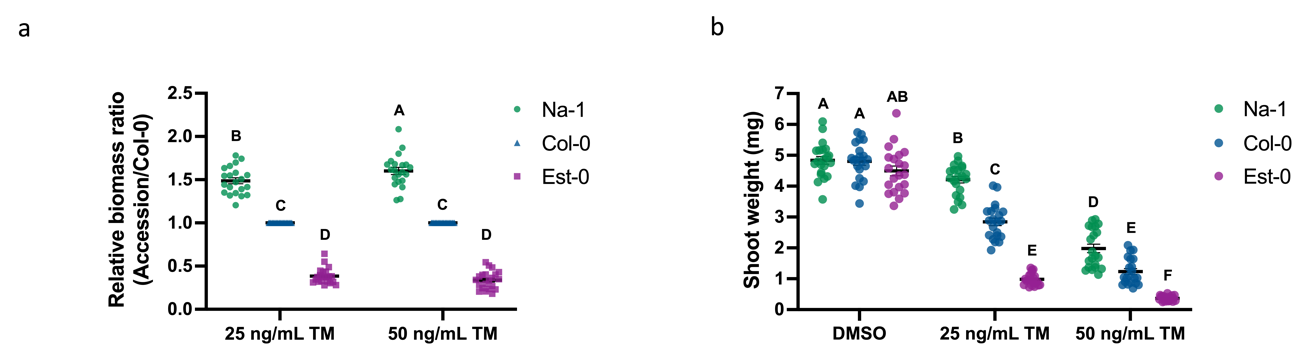


**Supplementary Figure 1. Shoot weight of Na-1, Col-0 and Est-0 accessions under chronic ER stress.** (a) Relative biomass ratio (RR) of Na-1 (green dots) or Est-0 (purple dots) compared to Col-0 (blue dots) was measured from seedlings germinated on 25 or 50 ng mL^-1^ TM or DMSO for 10 days. (b) Shoot weight of Na-1 (green dots), Col-0 (blue dots) and Est-0 (purple dots) seedlings germinated on media containing the indicated concentrations of TM or DMSO grown for 10 days. Data represent means ± SEM among twenty-one biological replicates from three independent experiments (n=21). Statistical significance was determined using a factorial linear mixed model framework followed by post-hoc testing using two-sided Tukey’s HSD test (multiple testing-controlled threshold used was *P*<0.05). Source data are provided as a Source Data file.


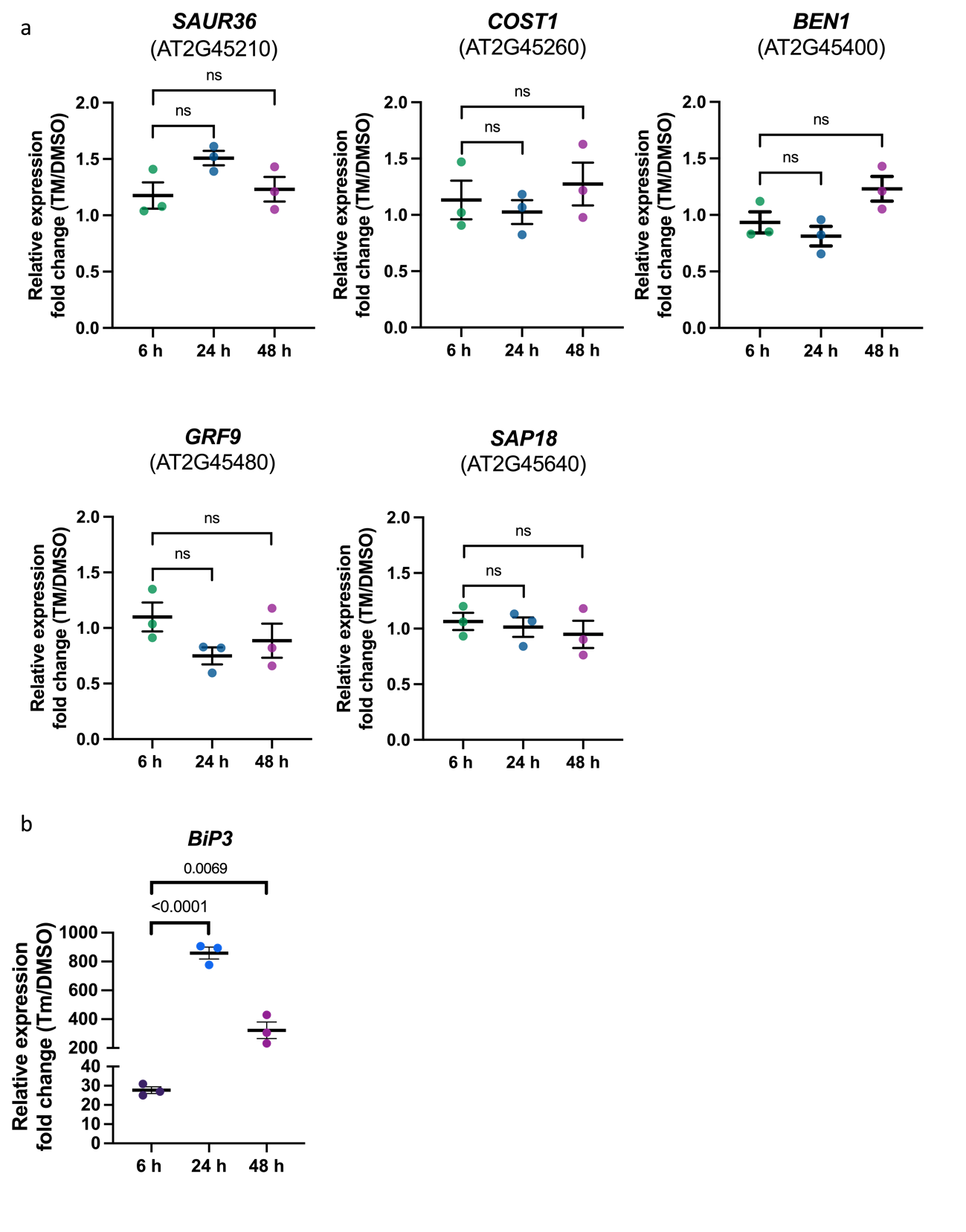


**Supplementary Figure 2. Expression of the selected genes in the regions of chromosome 2 identified in QTL mapping analyses with TM treatment.** (a) Transcript levels in 5-days-old Col-0 seedlings grown for 6, 24, and 48 hours in plates containing 1 µg mL^-1^ TM or DMSO analyzed by qRT-PCR. (b) *BiP3* transcript levels in 5-days-old Col-0 seedlings treated as indicated in (a) which was used as control of the UPR activation. *UBQ10* expression levels were used as an internal control. Values are presented relative to DMSO control and represent the mean ratio ± SEM among biological replicates (n=3). Statistical significance was determined by Student’s unpaired two-tailed *t*-test (*P*-value is shown in figure; ns, not significant). Source data are provided as a Source Data file.


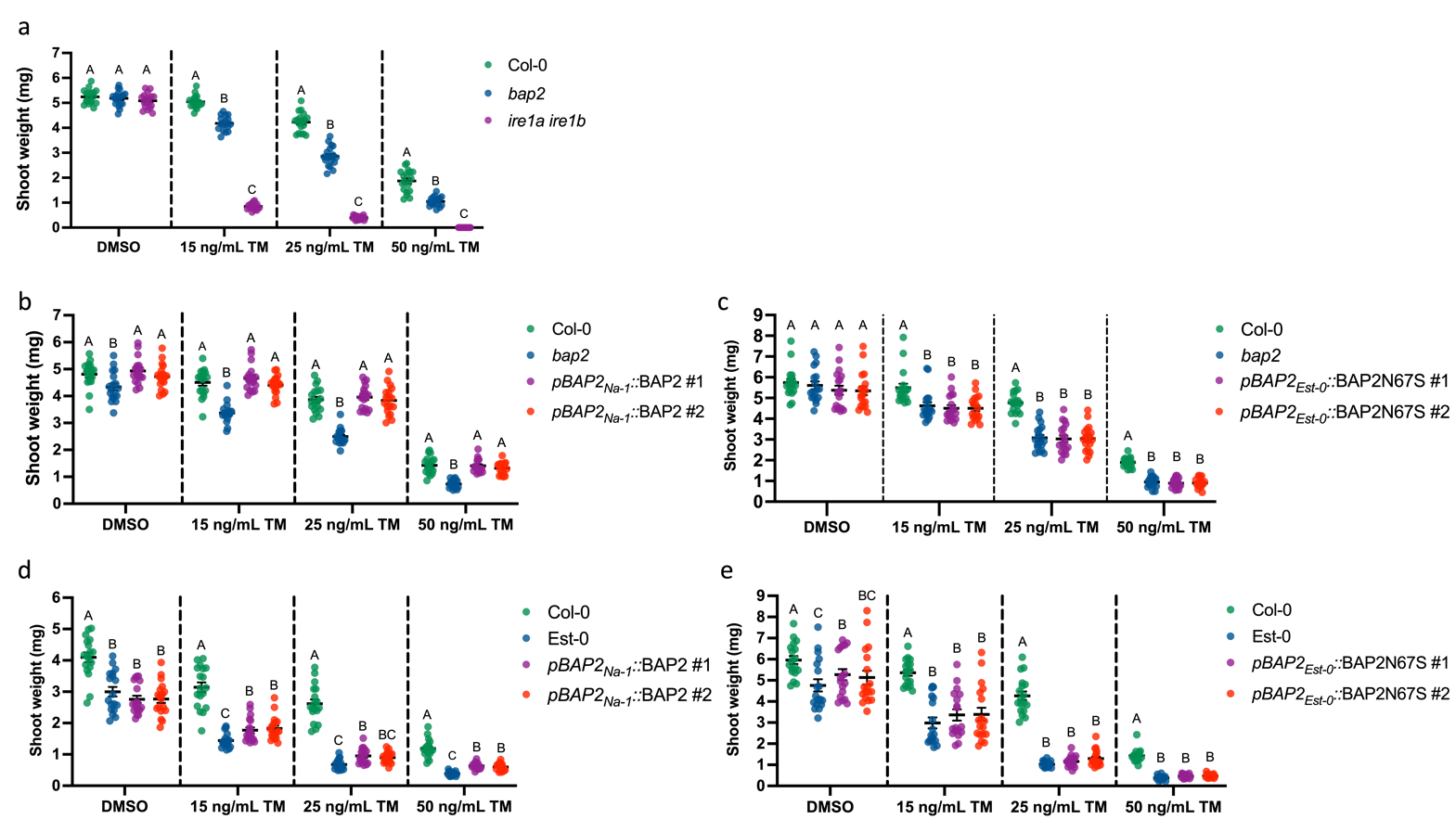


**Supplementary Figure 3. Raw shoot weight data from the chronic ER stress analyses.** (a) Shoot fresh weight of Col-0, *bap2* and *ire1a ire1b* seedlings germinated on media containing the indicated concentrations of TM or DMSO grown for 10 days. (b) Shoot fresh weight of the transgenic lines expressing *pBAP2_Na-1_*:BAP2 or (c) *pBAP2_Est-0_*:BAP2N67S in *bap-2* background treated as indicated in (a). (d) Shoot weight of Col-0, Est-0 and the transgenic lines expressing *pBAP2_Na-1_*:BAP2 or (e) *pBAP2_Est-0_*:BAP2N67S in Est-0 background treated as indicated in (a). Data represent means ± SEM among biological replicates (n=18). Statistical significance was determined using a factorial linear mixed model framework followed by post-hoc testing using two-sided Tukey’s HSD test (multiple testing-controlled threshold used was *P*<0.05). Source data are provided as a Source Data file.


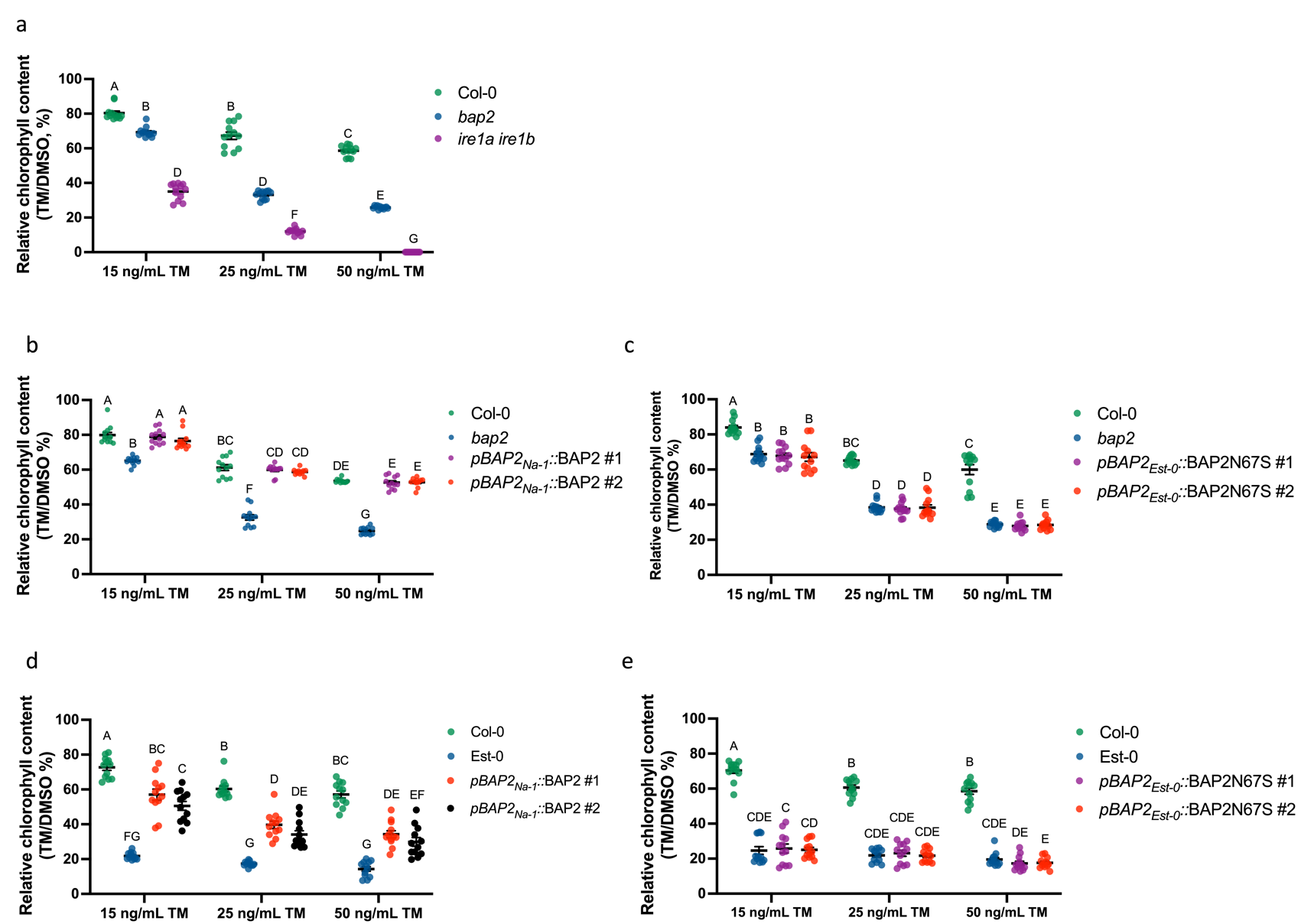


**Supplementary Figure 4. Relative chlorophyll content analysis in *bap2, pBAP2_Na-1_*:BAP2 and *pBAP2_Est-0_*:BAP2N67S transgenic lines in *bap2* background, and Est-0, *pBAP2_Na-1_*:BAP2 and *pBAP2_Est-0_*:BAP2N67S transgenic lines in Est-0 background under chronic ER stress.** (a) Relative chlorophyll content in Col-0, *bap2,* and *ire1a ire1b* seedlings germinated on media containing the indicated concentrations of TM or DMSO grown for 10 days. (b-c) Relative chlorophyll content of transgenic lines expressing *pBAP2_Na-1_*:BAP2 (b) or *pBAP2_Est-0_*:BAP2N67S (c) in *bap2* background treated as indicated in (a). (d-e) Relative chlorophyll content of transgenic lines expressing *pBAP2_Na-1_*:BAP2 (d) or *pBAP2_Est-0_*:BAP2N67S (e) in Est-0 background treated as indicated in (a). Data represent means ± SEM among biological replicates (n=12). Statistical significance was determined using a factorial linear mixed model framework followed by post-hoc testing using two-sided Tukey’s HSD test (multiple testing-controlled threshold used was *P*<0.05). Source data are provided as a Source Data file.


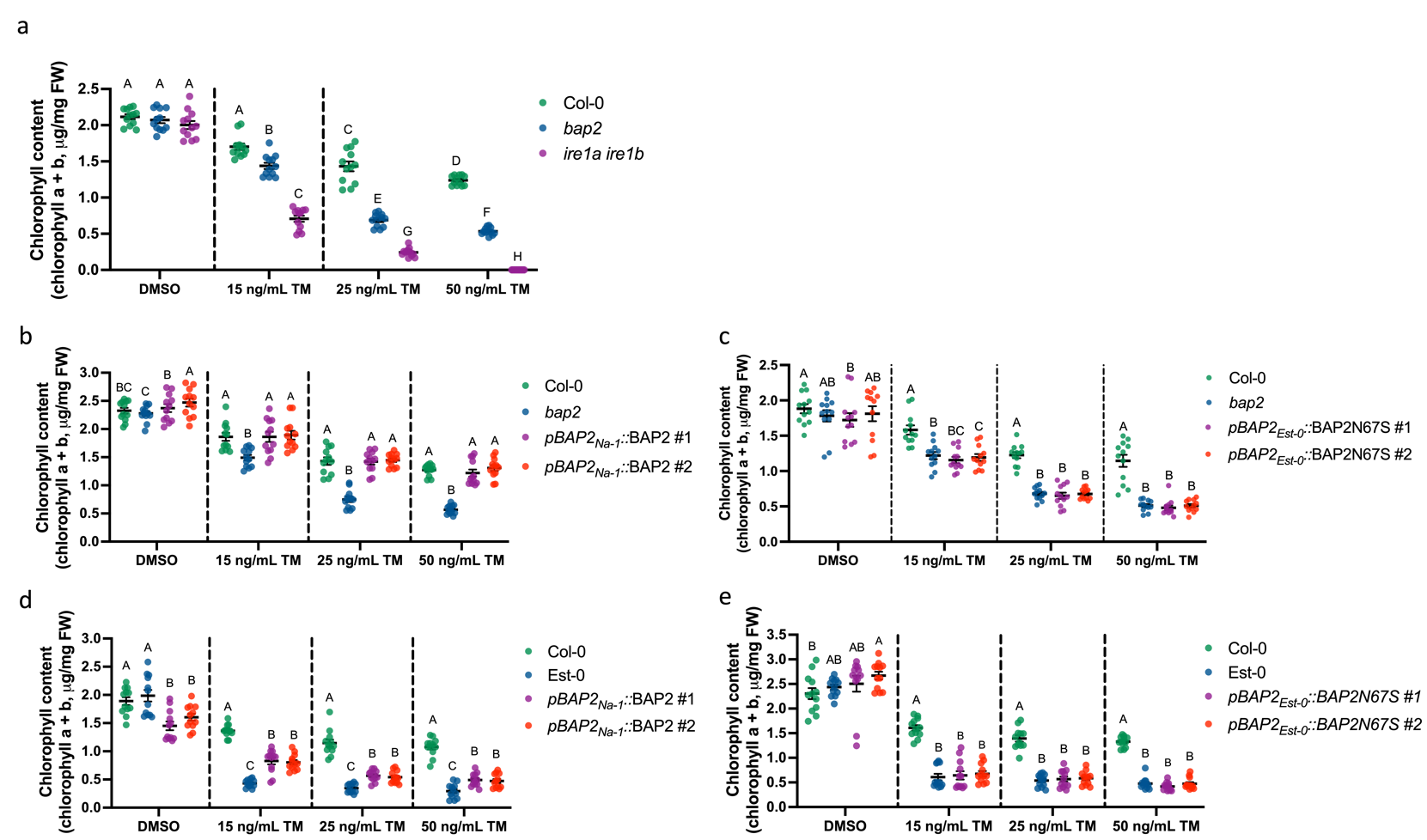


**Supplementary Figure 5. Raw data of the chlorophyll content analysis in *bap2, pBAP2_Na-1_*:BAP2 and *pBAP2_Est-0_*:BAP2N67S transgenic lines under chronic ER stress.** (a) Chlorophyll content in Col-0, *bap2,* and *ire1a ire1b* seedlings germinated on media containing the indicated concentrations of TM or DMSO grown for 10 days. (b-c) Chlorophyll content of transgenic lines expressing *pBAP2_Na-1_*:BAP2 (b) or *pBAP2_Est-0_*:BAP2N67S (c) in *bap2* background treated as indicated in (a). (d-e) Chlorophyll content of transgenic lines expressing *pBAP2_Na-1_*:BAP2 (d) or *pBAP2_Est-0_*:BAP2N67S in Est-0 background (e) treated as indicated in (a). Data represent means ± SEM among biological replicates (n=12). Statistical significance was determined using a factorial linear mixed model framework followed by post-hoc testing using two-sided) Tukey’s HSD test (multiple testing-controlled threshold used was *P*<0.05). Source data are provided as a Source Data file.


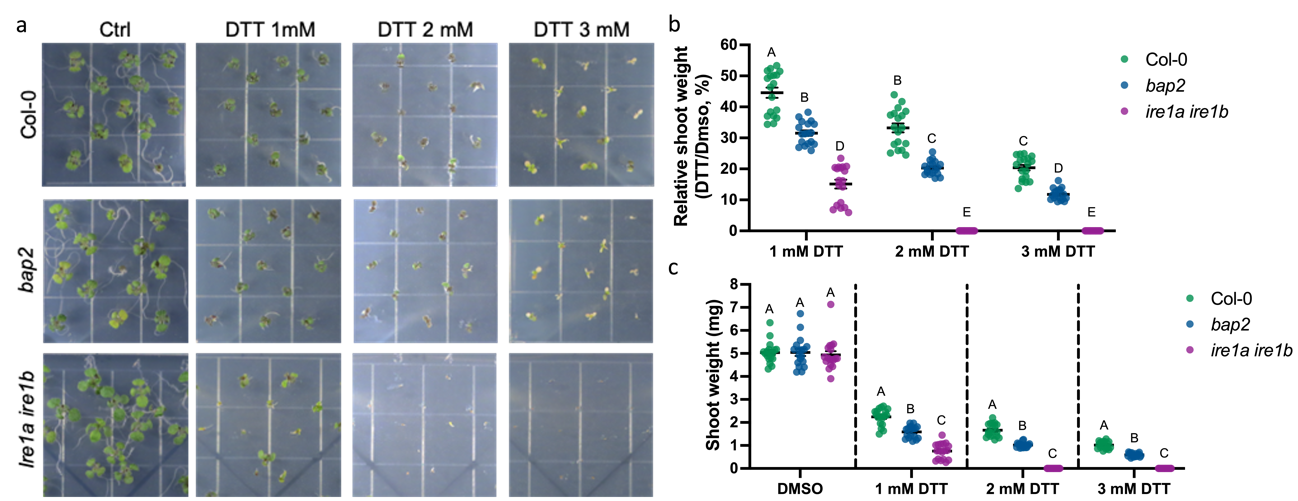


**Supplementary Figure 6. *bap2* loss-of-function mutant shows an increased sensitivity to DTT-induced ER stress.** (a) Representative Col-0, *bap2,* and *ire1a ire1b* seedlings germinated on media containing mock or the indicated concentrations of DTT grown for 10 days. (b) Relative shoot fresh weight and (c) shoot fresh weight of seedlings treated as indicated in (a). Data represent means ± SEM among biological replicates (n=18). Statistical significance was determined using a factorial linear mixed model framework followed by post-hoc testing using two-sided Tukey’s HSD test (multiple testing-controlled threshold used was *P*<0.05). Source data are provided as a Source Data file.


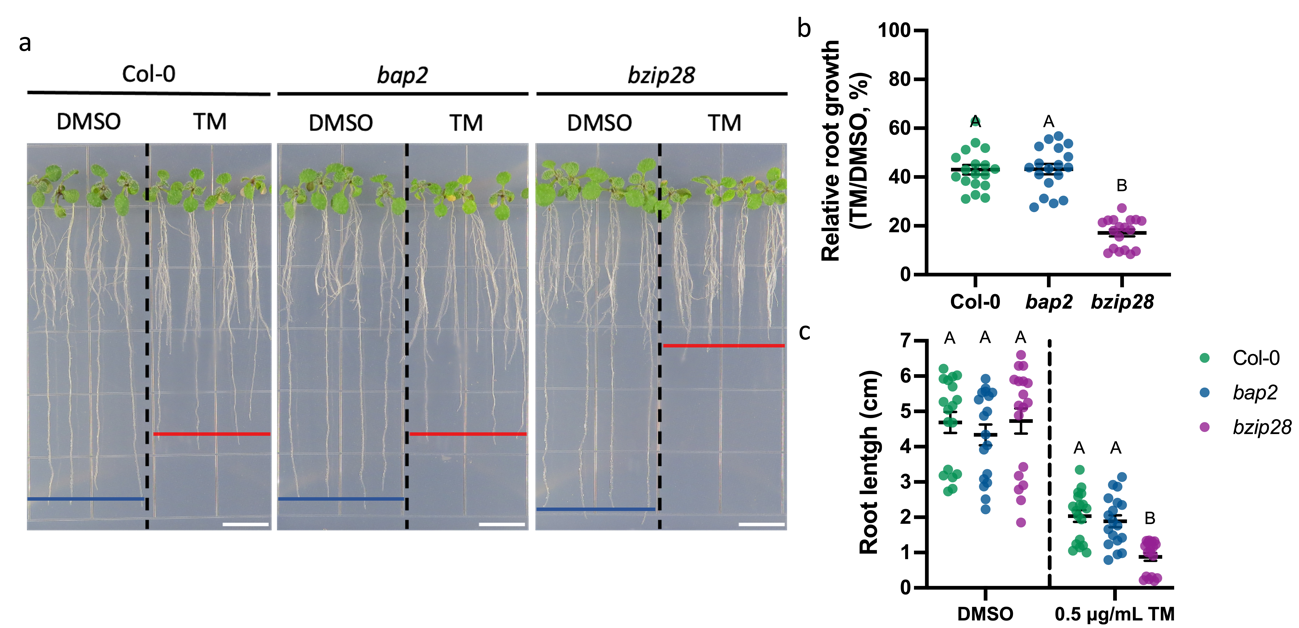


**Supplementary Figure 7. BAP2 is dispensable for ER stress recovery**. (a) Representative Col-0, *bzip28* and *bap2* seedlings grown on mock medium for 4 days from drug wash-out after 6 h pulse treatment with 0.5 𝜇g mL^-1^ TM or DMSO. (*b*) Relative primary root growth and (c) primary root growth of seedlings treated as indicated in (a). Data represent means ± SEM among biological replicates (n=18). Statistical significance was determined using a factorial linear mixed model framework followed by post-hoc testing using two-sided Tukey’s HSD test (multiple testing-controlled threshold used was *P*<0.05). (Scale bar = 1 cm). Source data are provided as a Source Data file.


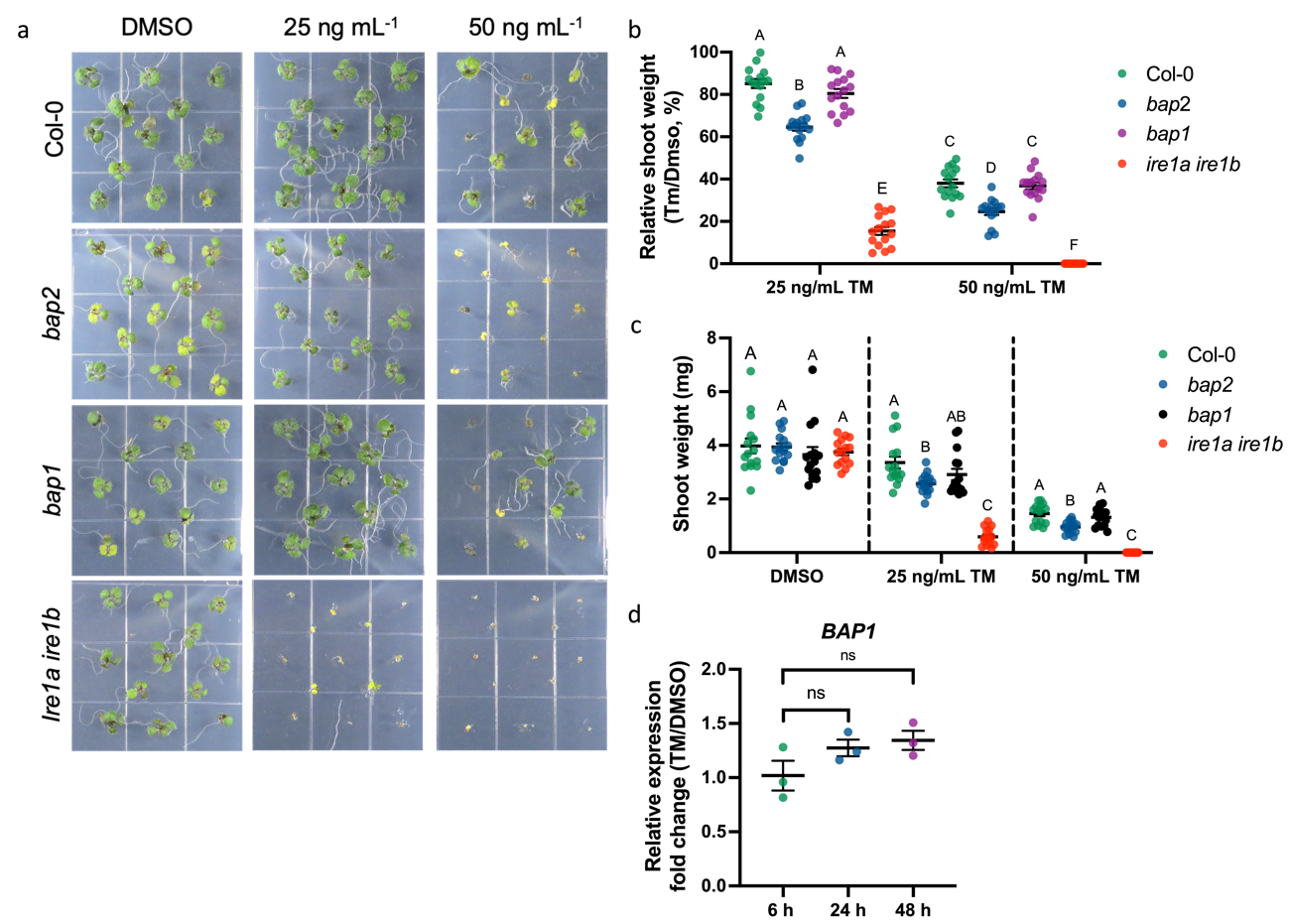


**Supplementary Figure 8. *bap1* mutant does not show the ER stress sensitive phenotype of *bap2*.** (a) Representative Col-0, *bap2, bap1*, and *ire1a ire1b* seedlings germinated on media containing the indicated concentrations of Tm or DMSO grown for 10 days. (b) Relative fresh shoot weight and (c) shoot fresh weight of seedling treated as indicated in (a). Data represent means ± SEM among biological replicates (n=15). Statistical significance was determined using a factorial linear mixed model framework followed by post-hoc testing using two-sided Tukey’s HSD test (multiple testing-controlled threshold used was *P*<0.05). (d) qRT-PCR analyses of *BAP1* expression in Col-0 upon 6, 24, and 48 hours of treatment with 1 µg mL^-1^ TM or DMSO. Values are presented relative to the indicated DMSO control. Transcription of *UBQ10* was used as an internal control. Data represent mean ± SEM among biological replicates (n=3). Statistical significance was determined by Student’s unpaired two-tailed *t*-test (ns, not significant). Source data are provided as a Source Data file.


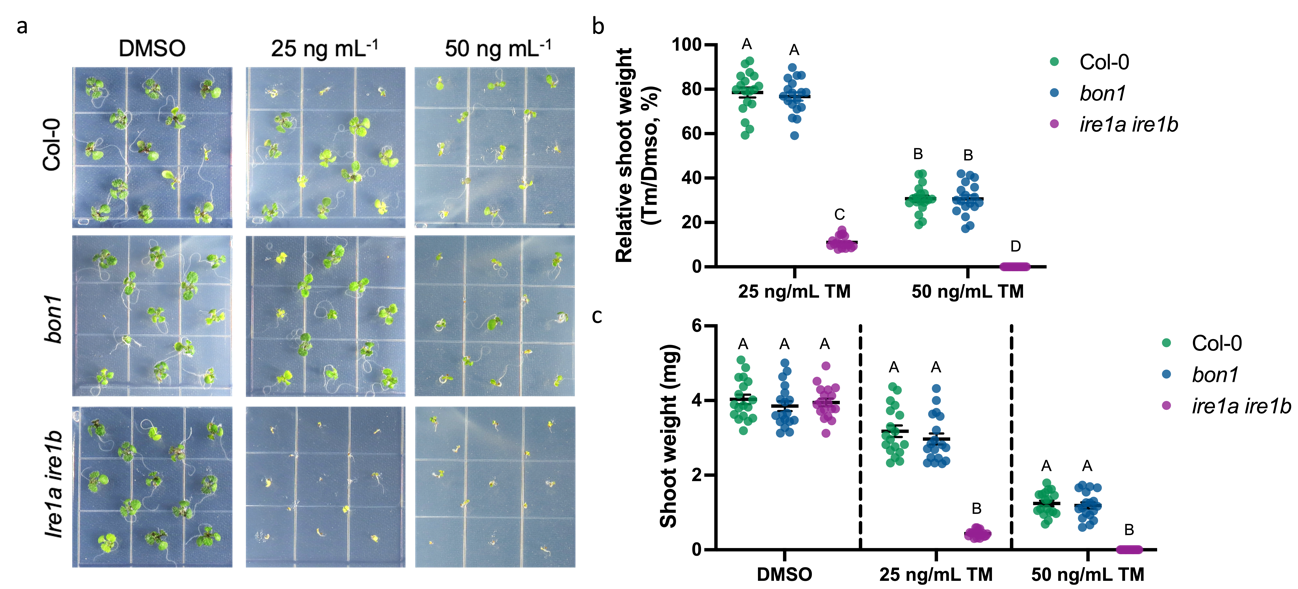


**Supplementary Figure 9. *bon-1* mutant does not show ER stress sensitive phenotype.** (a) Representative Col-0, *bon1*, and *ire1a ire1b* seedlings germinated on media containing the indicated concentrations of TM or DMSO grown for 10 days. (b) Relative fresh shoot weight or (c) shoot fresh weight of seedling treated as indicated in (a). Data represent means ± SEM among biological replicates (n=18). Statistical significance was determined using a factorial linear mixed model framework followed by post-hoc testing using two-sided Tukey’s HSD test (multiple testing-controlled threshold used was *P*<0.05). Source data are provided as a Source Data file.


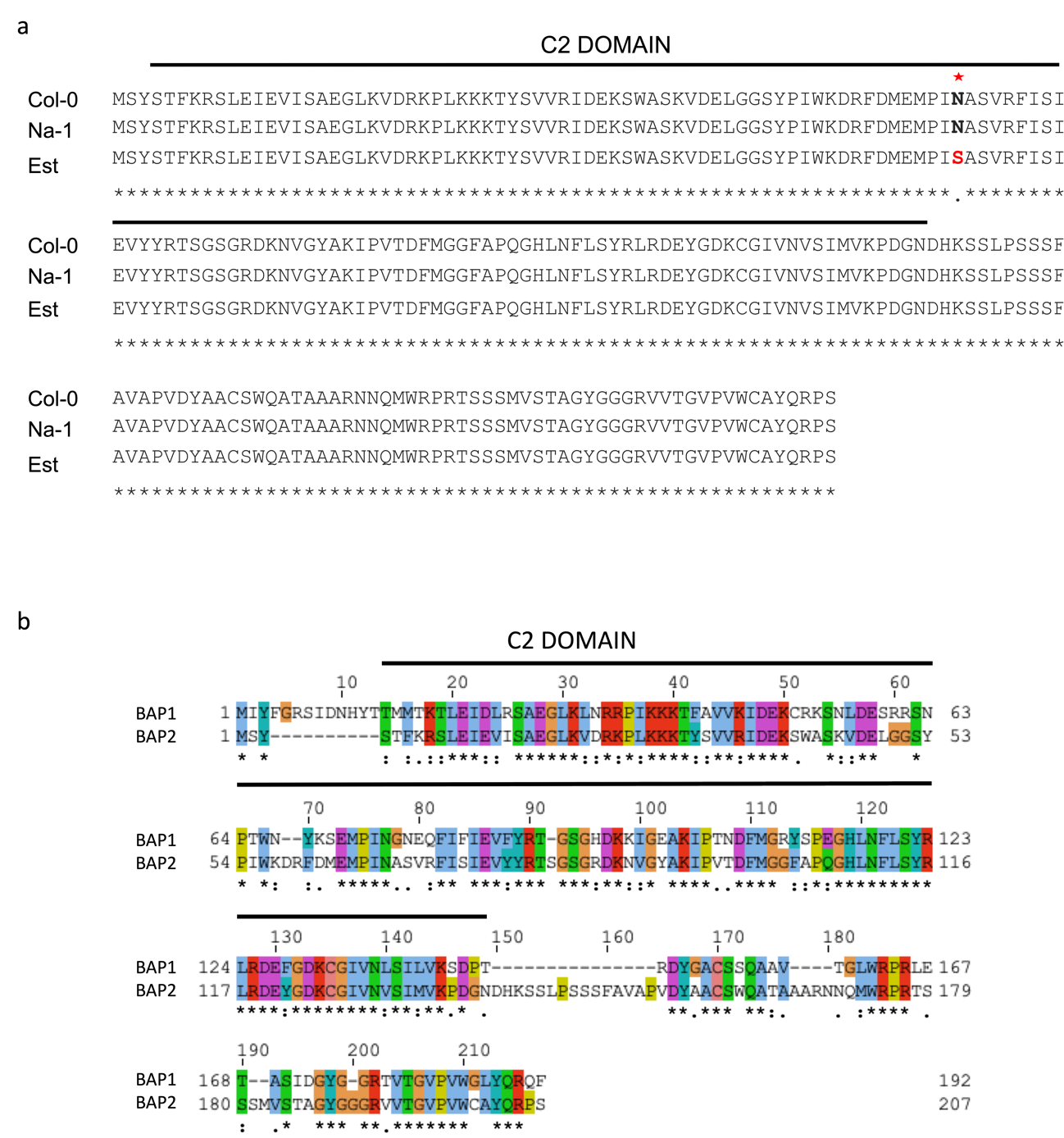


**Supplementary Figure 10. Alignments of Arabidopsis BAP proteins.** (a) Alignment of BAP2 proteins from the different accessions used in this study. (b) Alignment of Col-0 BAP1 and BAP2 proteins. Alignments were performed using T-coffee (<https://www.ebi.ac.uk/Tools/msa/tcoffee/>) and visualized by Jalview. Residues are colored following Clustal X criteria. Extension of C2 domain is indicated with a black line. Asterisks indicate conserved amino acids and red asterisks indicate position of the Est-0 non-synonymous mutation.


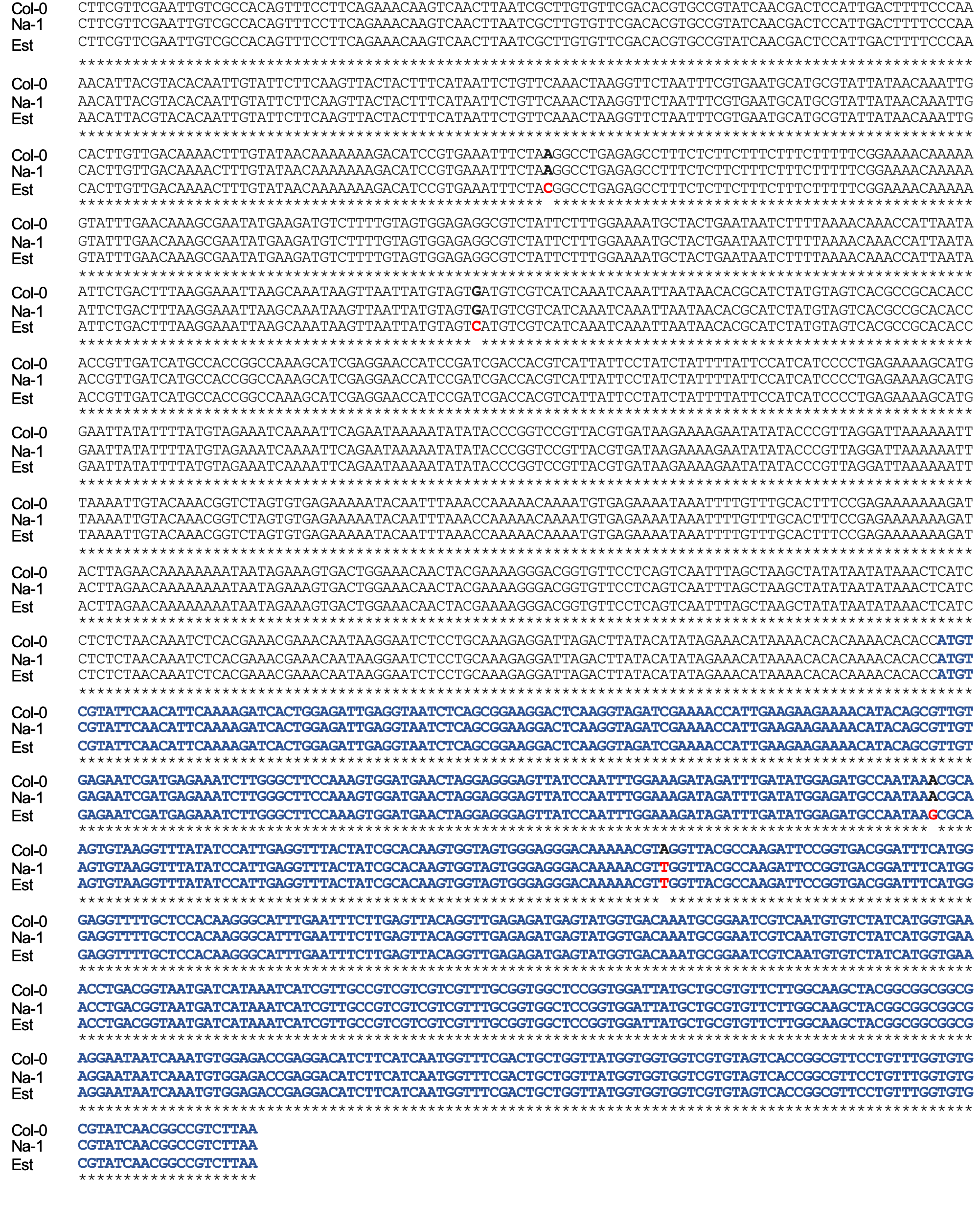


**Supplementary Figure 11. Multiple alignments of genomic *BAP2* gene including 1 kb promoter sequence among the Arabidopsis accessions**. Sequences of *BAP2* from Col-0, Na-1 and Est were obtained and aligned using T-Coffee (http://<https://www.ebi.ac.uk/Tools/msa/tcoffee/>). SNPs are indicated in red. Asterisks indicate conserved nucleotides.


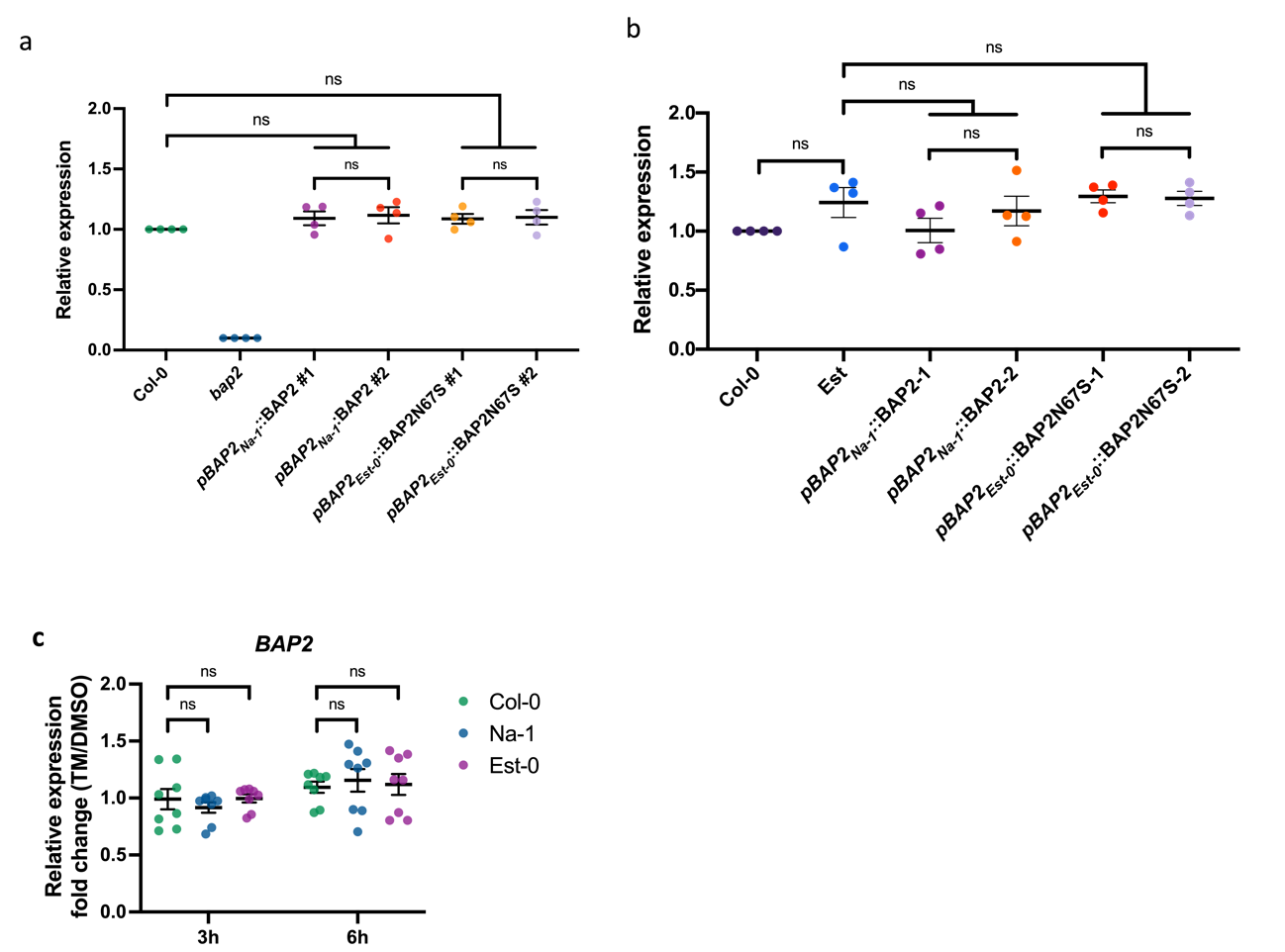


**Supplementary Figure 12. *BAP2* expression analysis in the transgenic lines under normal growth conditions and in the parental accessions under short-term ER stress.** (a) qRT-PCR analyses of *BAP2* expression in Col-0, *bap2*, and transgenic lines expressing Na-1 (*pBAP2_Na-1_*:BAP2) or Est-0 (*pBAP2_Est-0_*:BAP2N67S) genomic fragment (promoter and coding region) in *bap2* background under normal growth conditions. Values are presented relative to the indicated DMSO control. Transcription of *UBQ10* was used as an internal control. Data represent mean ± SEM among biological replicates (n=4). (b) qRT-PCR analyses of *BAP2* expression in Col-0, Est-0, and transgenic lines expressing Na-1 (*pBAP2_Na-1_*:BAP2) or Est-0 (*pBAP2_Est-0_*:BAP2N67S) genomic fragment (promoter and coding region) in Est-0 background under normal growth conditions. Values are presented relative to the indicated DMSO control. Transcription of *GAPDH was* used as an internal control. Data represent mean ± SEM among biological replicates (n=4). (*c*) qRT-PCR analyses of *BAP2* expression in Col-0, Est-0, and Na-1 upon 3-h or 6-h pulse treatment with 0.5 𝜇g mL^-1^ TM or DMSO. Values are presented relative to the indicated DMSO control. Transcription of *GAPDH* was used as an internal control. No differences were observed in primer sequence among Est-0, Na-1 and Col-0 accessions based on the 1,001 Arabidopsis genome database^56^. Data represent mean ± SEM among biological replicates (n=8). Statistical significance was determined by Student’s unpaired two-tailed *t*-test (ns, not significant). Source data are provided as a Source Data file.


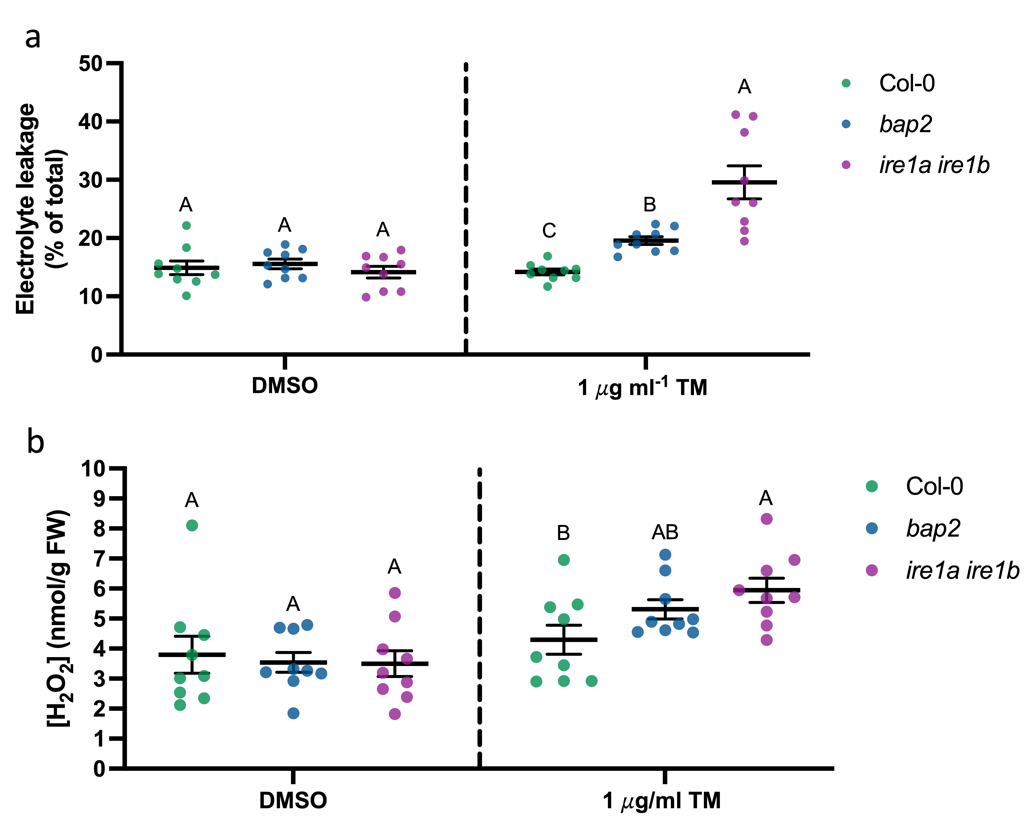


**Supplementary Figure 13. *BAP2* functions as a negative regulator of ER stress-induced cell death.** (a) Determination of the extent of cell death by quantification of percent electrolyte leakage or (b) determination of the accumulation of H_2_O_2_ quantified by an Amplex Ultra Red assay in Col-0, *bap2*, and *ire1a ire1b* 7-days-old seedlings treated with 1 𝜇g mL^-1^ TM or DMSO for 48 hours. Statistical significance was determined using a factorial linear mixed model framework followed by post-hoc testing using two-sided Tukey’s HSD test (multiple testing-controlled threshold used was *P*<0.05). Source data are provided as a Source Data file.


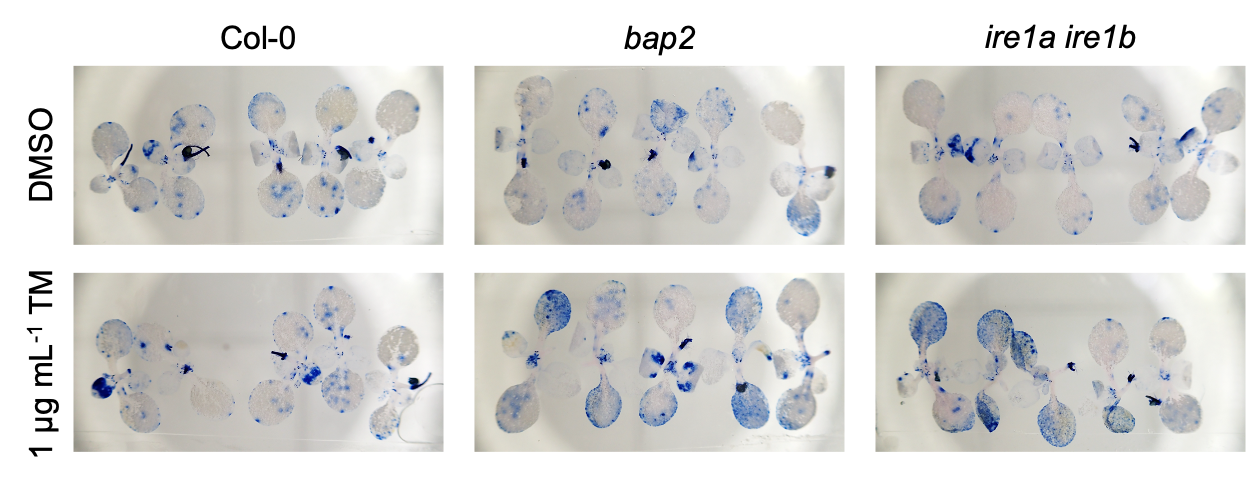


**Supplementary Figure 14. Determination of the extent of cell death by trypan blue staining.** Col-0, *bap2*, and *ire1a ire1b* 7-days-old seedlings treated with 1 𝜇g mL^-1^ TM or DMSO for 48 hours were stained using a trypan blue staining solution.


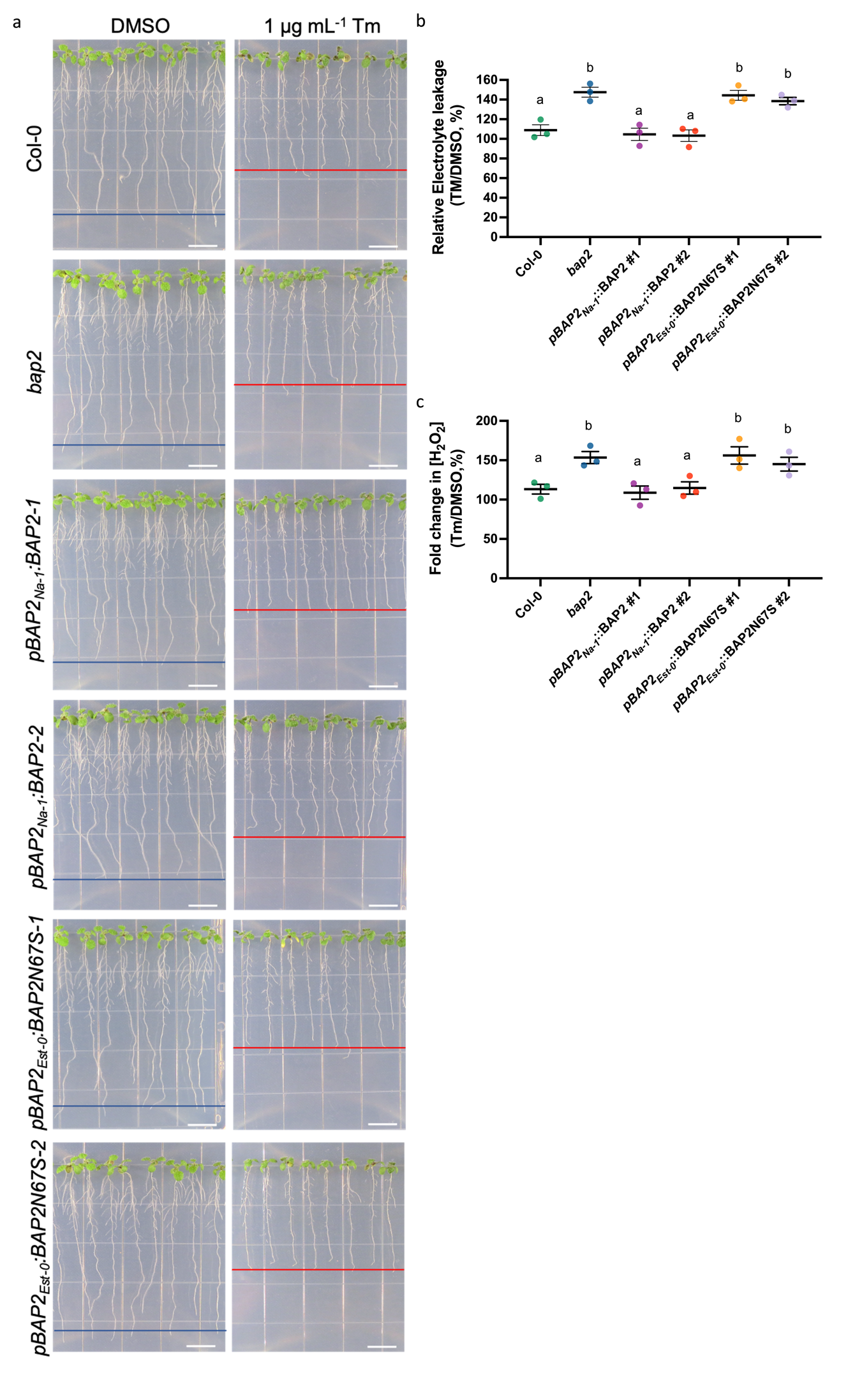


**Supplementary Figure 15. Transgenic lines expressing either Na-1 (*pBAP2_Na-1_*:BAP2) or Est-0 (*pBAP2_Est-0_*:BAP2N67S) genomic fragment in *bap2* background used for H_2_O_2_ and electrolyte leakage quantification.** (a) Representative Col-0, *bap2*, *pBAP2_Na-1_*:BAP2 and *pBAP2_Est-0_*:BAP2N67S 7-days-old seedlings treated with 1 𝜇g mL^-1^ TM or DMSO for 48 hours, which were used to determinate the extent of cell death by quantification of percent electrolyte leakage (b) or the accumulation of H_2_O_2_ quantified by an Amplex Ultra Red assay (c). Data represent mean ± SEM among biological replicates (n=3). Statistical significance was determined using a factorial linear mixed model framework followed by post-hoc testing using two-sided Tukey’s HSD test (multiple testing-controlled threshold used was *P*<0.05). (Scale bar = 1 cm). Source data are provided as a Source Data file.


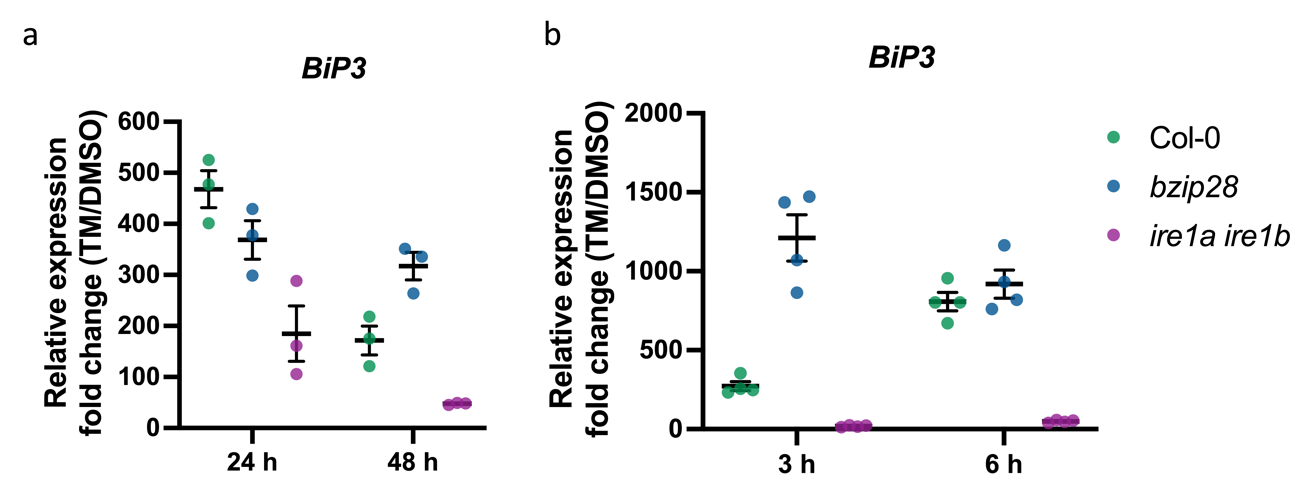


**Supplementary Figure 16. Induction of the expression of the ER stress marker *BiP3* during TM treatment**. (a) qRT-PCR analyses of *BiP3* expression in Col-0, *bzip28,* and *ire1a ire1b* seedlings after 24, and 48 hours upon 6-h pulse treatment with 0.5 µg mL^-1^ TM or DMSO. Values are presented relative to the indicated DMSO control. Transcription of *UBQ10* was used as an internal control. Data represent mean ± SEM among biological replicates (n=3). (b) qRT-PCR analyses of *BiP3* expression in Col-0, *bzip28,* and *ire1a ire1b* after 3-h or 6-h pulse treatment with 0.5 𝜇g mL^-1^ TM or DMSO. Values are presented relative to the indicated DMSO control. Transcription of *UBQ10* was used as an internal control. Data represent mean ± SEM among among biological replicates (n=4). Source data are provided as a Source Data file.

**Supplementary Figure 17. *IRE1A* and *IRE1B* transcription levels are not induced in the *bap2* mutant.** qRT-PCR analyses of *BiP3* expression in Col-0 and *bap2* after 6-h pulse treatment with 0.5 𝜇g mL^-1^ TM or DMSO. Values are presented relative to the indicated DMSO control. Transcription of *UBQ10* was used as an internal control. Data represent mean ± SEM among biological replicates (n=8). Statistical significance was determined by Student’s unpaired two-tailed *t*-test (ns, not significant). Source data are provided as a Source Data file.


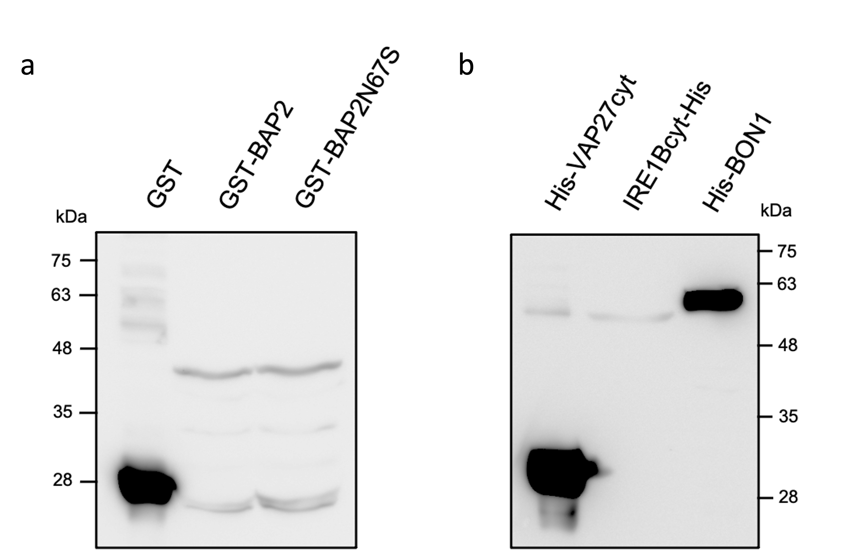


**Supplementary Figure 18. In-puts used for *in vitro* interaction assay.** (a) GST, GST-BAP2 and GST-BAP2N67S protein extracts or (b) IRE1B-His, BON1-His and His-VAP27cyto protein extracts were obtained from E.coli BL2. The same amount of protein from each protein extracts was loaded on a 10% SDS-page gel. Western blot analysis was performed using anti-GST monoclonal antibody for GST, GST-BAP2 and GST-BAP2NG7S extracts (a) or anti-His monoclonal antibody for His-VAP2cyt, IRE1Bcyt-His and His-BON1 (b). The experiment was repeated three times with similar results.


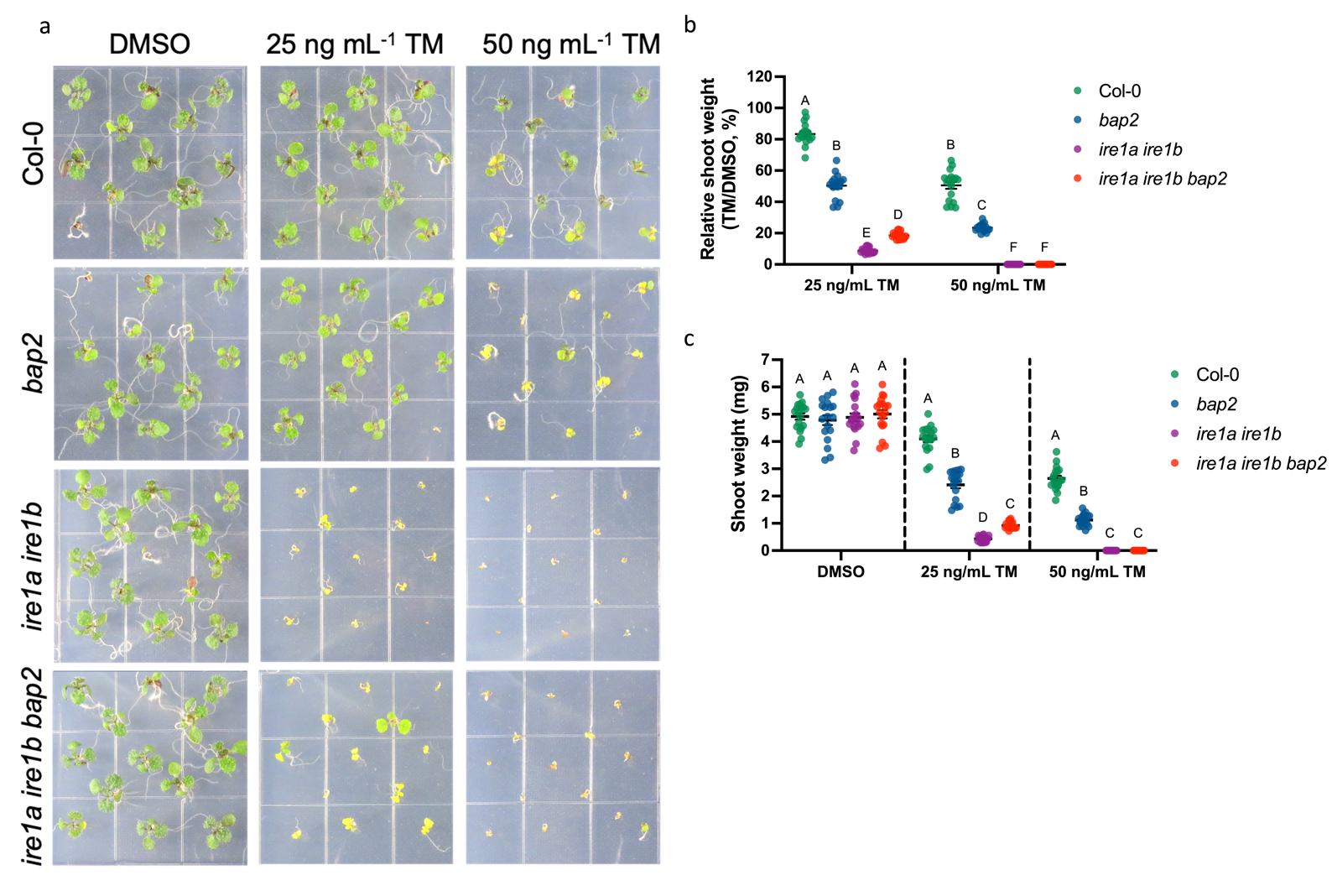


**Supplementary Figure 19. *Ire1a ire1b bap2* shows a reduced sensitivity to TM compared to *ire1a ire1b* at low TM concentrations under chronic stress.** (a) Representative Col-0, *bap2,* *ire1a ire1b* and *ire1a ire1b bap2* seedlings germinated on media containing the indicated concentrations of TM or DMSO and grown for 10 days. (b) Relative shoot fresh weight of Col-0, *bap2,* *ire1a ire1b* and *ire1a ire1b bap2* seedlings treated as indicated in (a). (c) Shoot fresh weight of Col-0, *bap2,* *ire1a ire1b* and *ire1a ire1b bap2* seedlings treated as indicated in (a). Data represent means ± SEM among biological replicates (n=18). Data represent means ± SEM among biological replicates (n=18). Statistical significance was determined using a factorial linear mixed model framework followed by post-hoc testing using two-sided Tukey’s HSD test (multiple testing-controlled threshold used was *P*<0.05). Source data are provided as a Source Data file.


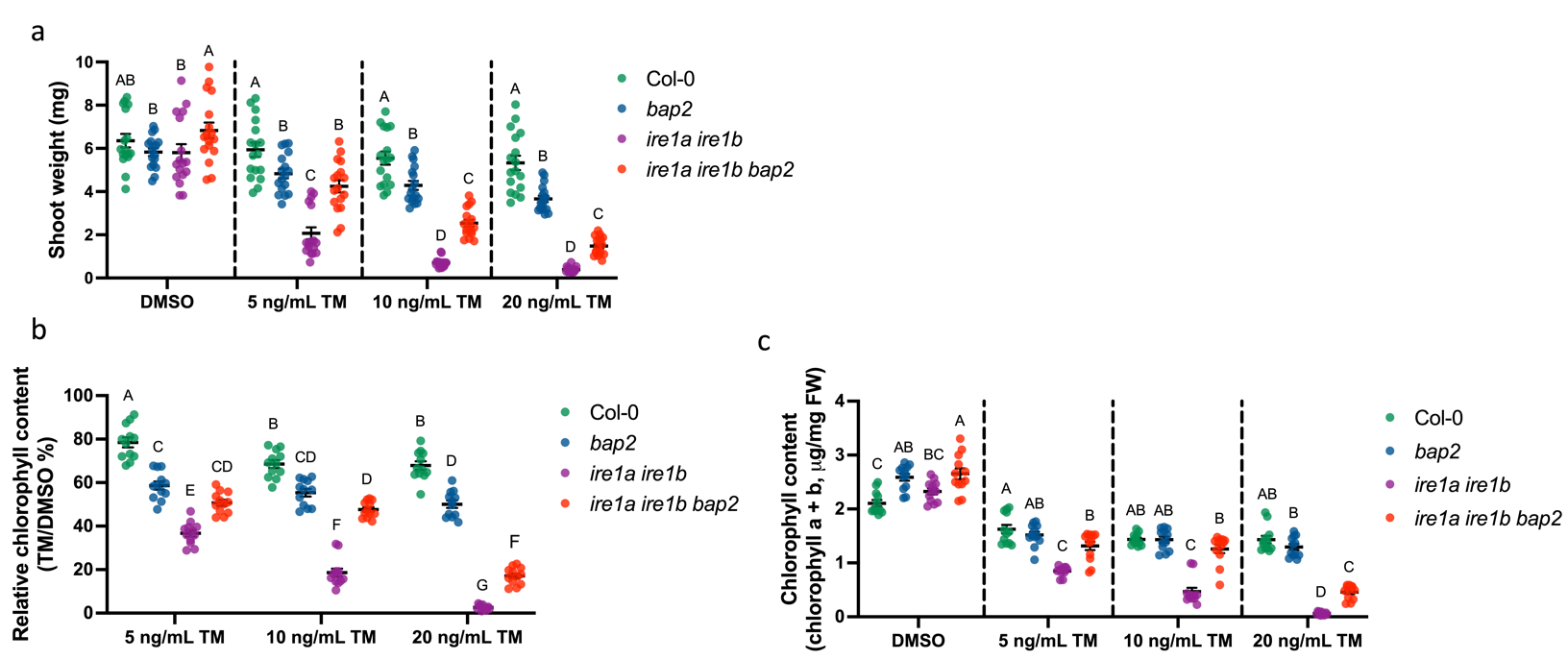


**Supplementary Figure 20. *Ire1a ire1b bap2* reduces *ire1a ire1b* sensitivity to chronic ER stress.** (a) Shoot fresh weight of Col-0, *bap2, ire1a ire1b* and *ire1a ire1b bap2* seedlings germinated on media containing the indicated concentrations of TM or DMSO and grown for 10 days. Data represent means ± SEM among biological replicates (n=18). (b) Relative chlorophyll content and (c) total chlorophyll content of Col-0, *bap2,* *ire1a ire1b* and *ire1a ire1b bap2* seedlings treated as indicated in (a). Data represent means ± SEM among biological replicates (n=12). Statistical significance was determined using a factorial linear mixed model framework followed by post-hoc testing using two-sided Tukey’s HSD test (multiple testing-controlled threshold used was *P*<0.05). Source data are provided as a Source Data file.

**Supplementary Figure 21. *Ire1a ire1b bap2* and *ire1a ire1b* show similar H_2_O_2_ accumulation during chronic ER stress.** Relative percent of H_2_O_2_ accumulation quantified by an Amplex Ultra Red assay in Col-0, *bap2, ire1a ire1b* and *ire1a ire1b bap2* seedlings germinated on media containing the indicated concentrations of TM or DMSO and grown for 10 days. Data represent mean ± SEM among biological replicates (n=9). Statistical significance was determined using a factorial linear mixed model framework followed by post-hoc testing using two-sided Tukey’s HSD test (multiple testing-controlled threshold used was *P*<0.05). Source data are provided as a Source Data file.


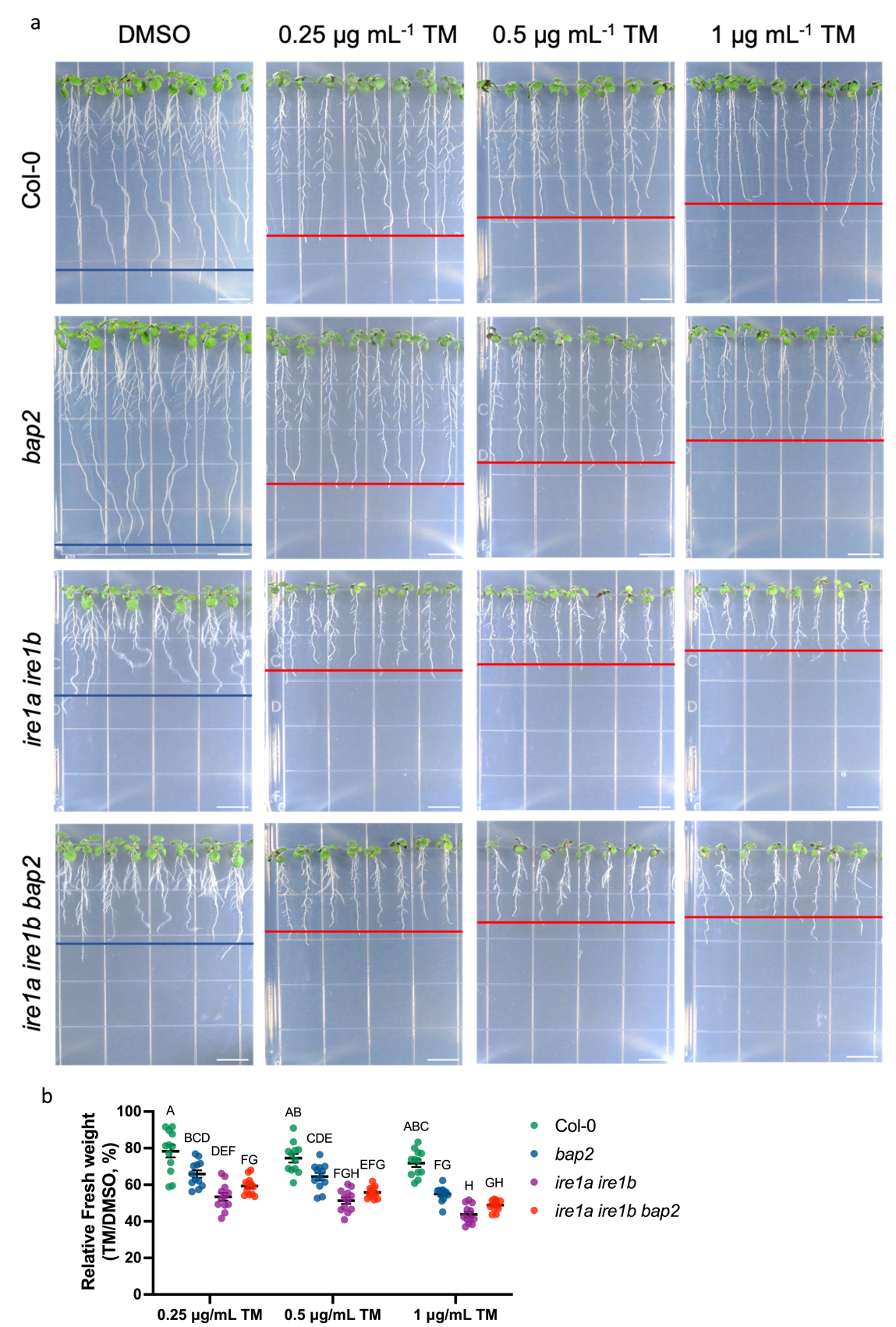


**Supplementary Figure 22. Col-0, *bap2*, *ire1a ire1b* and *ire1a ire1b bap2* seedlings used for H_2_O_2_ accumulation and electrolyte leakage quantification during prolonged ER stress.** (a) Representative Col-0, *bap2*, *ire1a ire1b* and *ire1a ire1b bap2* 7-days-old seedlings treated with the indicated TM or DMSO concentration for 48 hours that were used to determine the extent of cell death by quantification of the accumulation of H_2_O_2_ as quantified by an Amplex Ultra Red assay (Figure 6d) or percent electrolyte leakage (Figure 6e). (b) Relative fresh weight of Col-0, *bap2*, *ire1a ire1b* and *ire1a ire1b bap2* 7-days-old seedlings treated as indicated in (a). Data represent means ± SEM among biological replicates (n=9). Statistical significance was determined using a factorial linear mixed model framework followed by post-hoc testing using two-sided Tukey’s HSD test (multiple testing-controlled threshold used was *P*<0.05). Source data are provided as a Source Data file.


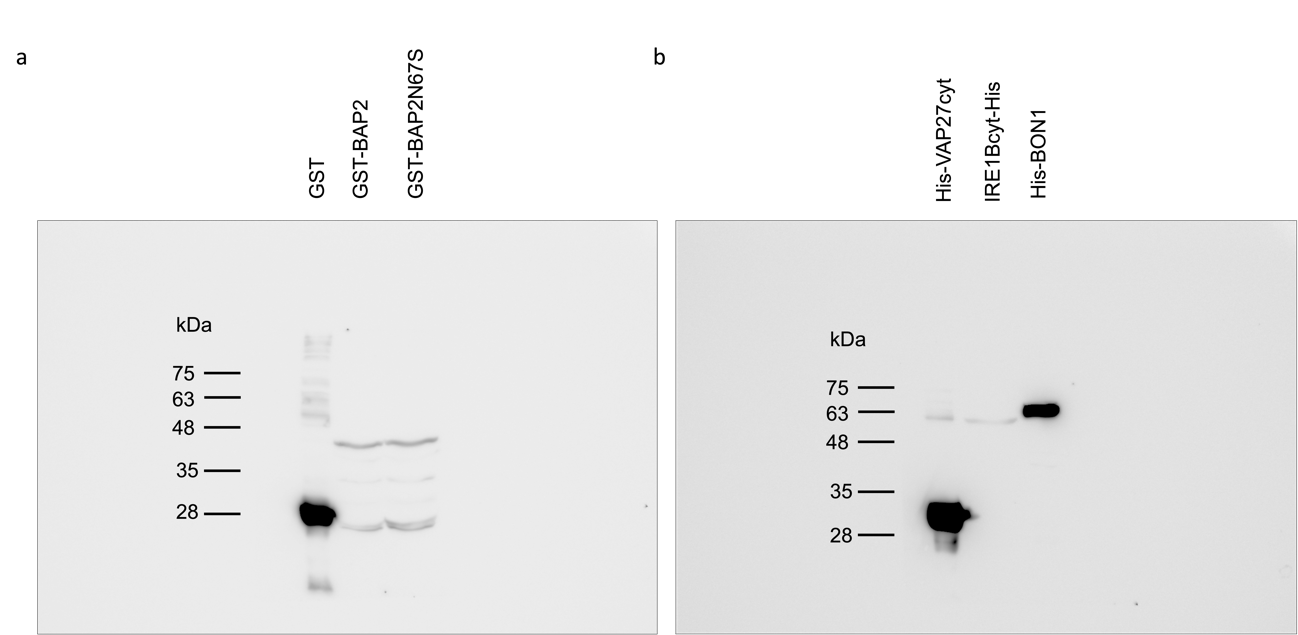


**Supplementary Figure 23. Uncropped blots from Supplementary Figure 18.** (a) GST, GST-BAP2 and GST-BAP2N67S protein extracts or (b) IRE1B-His, BON1-His and His-VAP27cyto protein extracts were obtained from E.coli BL2. The same amount of protein from each protein extracts was loaded on a 10% SDS-page gel. Western blot analysis was performed using anti-GST monoclonal antibody for GST, GST-BAP2 and GST-BAP2NG7S extracts (a) or anti-His monoclonal antibody for His-VAP2cyt, IRE1Bcyt-His and His-BON1 (b).
