# Supplementary material for: Programmed cell death regulator BAP2 is required for IRE1-mediated unfolded protein response in Arabidopsis
Source: Nat Commun. 2024 Jul 10;15:5804. doi: 10.1038/s41467-024-50105-6 (PMC11237027; doi:10.1038/s41467-024-50105-6)
Supplement: Supplementary file 3 — Description of Additional Supplementary Files [file 41467_2024_50105_MOESM3_ESM.pdf]

Description of Additional Supplementary Files

Supplementary Data 1. Relative Ratio of the 350 A. thaliana accessions used in this work.

Supplementary Data 2. Relative Ratio of 400 F3 lines used in this work

Supplementary Data 3. QTL analysis results (SNP-index and ΔSNP-index).

Supplementary Data 4. Genes identified within the chromosomal confidence interval identified in Chr 2.

Supplementary Data 5. Primers used in this work.
