# Supplementary material for: Programmed cell death regulator BAP2 is required for IRE1-mediated unfolded protein response in Arabidopsis
Source: Nat Commun. 2024 Jul 10;15:5804. doi: 10.1038/s41467-024-50105-6 (PMC11237027; doi:10.1038/s41467-024-50105-6)
Supplement: Supplementary file 9 — Reporting Summary [file 41467_2024_50105_MOESM9_ESM.pdf]

nature portfolio

Corresponding author(s): fb@msu.edu

Last updated by author(s): 6/19/2024

Reporting Summary

Nature Portfolio wishes to improve the reproducibility of the work that we publish. This form provides structure for consistency and transparency in reporting. For further information on Nature Portfolio policies, see our Editorial Policies and the Editorial Policy Checklist.

Statistics

For all statistical analyses, confirm that the following items are present in the figure legend, table legend, main text, or Methods section.

n/a	Confirmed	
☐	☒	The exact sample size (*n*) for each experimental group/condition, given as a discrete number and unit of measurement
☐	☒	A statement on whether measurements were taken from distinct samples or whether the same sample was measured repeatedly
☐	☒	The statistical test(s) used AND whether they are one- or two-sided<br>*Only common tests should be described solely by name; describe more complex techniques in the Methods section.*
☐	☒	A description of all covariates tested
☐	☒	A description of any assumptions or corrections, such as tests of normality and adjustment for multiple comparisons
☐	☒	A full description of the statistical parameters including central tendency (e.g. means) or other basic estimates (e.g. regression coefficient) AND variation (e.g. standard deviation) or associated estimates of uncertainty (e.g. confidence intervals)
☐	☒	For null hypothesis testing, the test statistic (e.g. *F*, *t*, *r*) with confidence intervals, effect sizes, degrees of freedom and *P* value noted<br>*Give P values as exact values whenever suitable.*
☒	☐	For Bayesian analysis, information on the choice of priors and Markov chain Monte Carlo settings
☒	☐	For hierarchical and complex designs, identification of the appropriate level for tests and full reporting of outcomes
☒	☐	Estimates of effect sizes (e.g. Cohen's *d*, Pearson's *r*), indicating how they were calculated

Our web collection on statistics for biologists contains articles on many of the points above.

Software and code

Policy information about availability of computer code

Data collection	Excel
Data analysis	For the accession analyses: SAS 9.4, the proc mixed procedure<br>For the QTL-seq: FastQC, cutadapt, bwa, samtools, bcftools and R following the method described in Takagi et al. 2013 - The Plant Journal.<br>For statistical analysis: JMP Pro 17 using linear mixed models

For manuscripts utilizing custom algorithms or software that are central to the research but not yet described in published literature, software must be made available to editors and reviewers. We strongly encourage code deposition in a community repository (e.g. GitHub). See the Nature Portfolio guidelines for submitting code & software for further information.

Data

Policy information about availability of data

All manuscripts must include a data availability statement. This statement should provide the following information, where applicable:

- Accession codes, unique identifiers, or web links for publicly available datasets
- A description of any restrictions on data availability
- For clinical datasets or third party data, please ensure that the statement adheres to our policy

All data supporting the findings of this study are available within this paper and its Supplementary Materials files. QTL data supporting the finding of this study have been deposited in the BioSample database and are accessible through the BioProject accession code PRJNA1125890. NA-1 and Est-0 WGS data have been deposited

in the Figshare database and are accessible through https://doi.org/10.6084/m9.figshare.24787494. QTL analysis data are provided in Supplementary Data 3 file. All the biological material generated in this work are available from the authors upon request. Source data are provided as a Source Data file.

Research involving human participants, their data, or biological material

Policy information about studies with human participants or human data. See also policy information about sex, gender (identity/presentation), and sexual orientation and race, ethnicity and racism.

Reporting on sex and gender	*Use the terms sex (biological attribute) and gender (shaped by social and cultural circumstances) carefully in order to avoid confusing both terms. Indicate if findings apply to only one sex or gender; describe whether sex and gender were considered in study design; whether sex and/or gender was determined based on self-reporting or assigned and methods used.*<br>*Provide in the source data disaggregated sex and gender data, where this information has been collected, and if consent has been obtained for sharing of individual-level data; provide overall numbers in this Reporting Summary. Please state if this information has not been collected.*<br>*Report sex- and gender-based analyses where performed, justify reasons for lack of sex- and gender-based analysis.*
Reporting on race, ethnicity, or other socially relevant groupings	*Please specify the socially constructed or socially relevant categorization variable(s) used in your manuscript and explain why they were used. Please note that such variables should not be used as proxies for other socially constructed/relevant variables (for example, race or ethnicity should not be used as a proxy for socioeconomic status).*<br>*Provide clear definitions of the relevant terms used, how they were provided (by the participants/respondents, the researchers, or third parties), and the method(s) used to classify people into the different categories (e.g. self-report, census or administrative data, social media data, etc.)*<br>*Please provide details about how you controlled for confounding variables in your analyses.*
Population characteristics	*Describe the covariate-relevant population characteristics of the human research participants (e.g. age, genotypic information, past and current diagnosis and treatment categories). If you filled out the behavioural & social sciences study design questions and have nothing to add here, write "See above."*
Recruitment	*Describe how participants were recruited. Outline any potential self-selection bias or other biases that may be present and how these are likely to impact results.*
Ethics oversight	*Identify the organization(s) that approved the study protocol.*

Note that full information on the approval of the study protocol must also be provided in the manuscript.

Field-specific reporting

Please select the one below that is the best fit for your research. If you are not sure, read the appropriate sections before making your selection.

[x] Life sciences [ ] Behavioural & social sciences [ ] Ecological, evolutionary & environmental sciences

For a reference copy of the document with all sections, see nature.com/documents/nr-reporting-summary-flat.pdf

Life sciences study design

All studies must disclose on these points even when the disclosure is negative.

Sample size	For the natural accession screen, we selected 350 different accessions based on their availability from public databases, geographical distribution, availability of fully or partially sequenced genomes and feasibility of conducting the experiments to detect the widest possible range of ER stress responses.<br>For the F3 population screen, we screened 400 F3 progeny. At that point, we knew very little about the genetic architecture underlying the tolerance difference between Na and Est-1. As such, we were limited as much by what could logistically be screened as by any informed decision. 400 is a pretty ambitious effort as it should represent a substantial amount of recombination between parental alleles and allow reasonable ability to differentiate tails of the phenotype. If the trait was highly polygenic, the QTL-seq approach would likely not have worked well. In the end, the 400 allowed at least a coarse localization as indicated in Figure 1.<br>For chronic and adaptive experiments: 13 seedlings were grown in each plate for each condition. This allowed us to grown four different genetic backgrounds at the same plate.
Data exclusions	We pre-establish that data coming from plates that showed any contamination were excluded since contamination could interfere in our phenotypic analysis. For the expression analysis in bap2 mutant, 2 samples from 12 collected samples were excluded from several genotypes in order to unify the number of samples to 10 samples. For the expression analysis set performed in bap2 bzip28 and bap2 bzip60, 9 samples were collected but one was excluded due to a possible contamination during its manipulation.
Replication	All the phenotypic screening analyses were performed at least three times with reproducible results. For the chronic and phenotypic analyzes, experiments were performed three times, and each time was based on 6 replicates, except for the bap1 analyses which were performed three times with 3 replicates each time. Of note, minimal variation among the bap1 samples was verified. Hence, although the number of biological replicates and technical replicates were different, we used the needed number of replicates to gain statistically meaningful data for all the genetic background.<br>The gene expression analyses were also performed three times. However, BAP2 and BAP1 chronic expression analysis that was performed twice since they were confirmed in our RNAseq database Ko et al., 2023 (doi: 10.1038/s41477-023-01480-3). The BAP2 expression analysis performed in the transgenic lines expressing pBAP2Na-1:BAP2, and pBAP2Est:BAP2N67S in bap2 or Est-0 background was performed once

since we did not observe significant differences between both independent lines. In addition, the PCD and ROS analysis were also performed three times, less the analysis performed in the transgenic lines expressing pBAP2Na-1:BAP2, and pBAP2Est:BAP2N67S in bap2 background which was performed once since we did not observe significant differences between both independent lines.

In-vitro interactions assays were performed four times, three the attemps were successful, less the first one due to a problem with the IPTG lot (protein induction was not observed).

Randomization

Plates were distributed randomly inside the growth chambers. For the accession screening, each accession was assigned a random number. Then, accessions were grouped in groups of 7 based on their numbering. Each group was germinated in the same plate together. As internal control, our reference accession, Col-0, was germinated on each plate in the phenotypic screenings and chronic phenotypic analyzes. In addition, to minimize the effect of the variation due to different plates, all the genotypes analyzed in each chronic phenotypic analysis were germinated in the same plate.

Blinding

In order to avoid any bias during the accession screen, each accession was assigned a random number which was used in each replicate.

Reporting for specific materials, systems and methods

We require information from authors about some types of materials, experimental systems and methods used in many studies. Here, indicate whether each material, system or method listed is relevant to your study. If you are not sure if a list item applies to your research, read the appropriate section before selecting a response.

Materials & experimental systems

n/a	Involved in the study
☐	☒ Antibodies
☒	☐ Eukaryotic cell lines
☒	☐ Palaeontology and archaeology
☒	☐ Animals and other organisms
☒	☐ Clinical data
☒	☐ Dual use research of concern
☐	☒ Plants

Methods

n/a	Involved in the study
☒	☐ ChIP-seq
☒	☐ Flow cytometry
☒	☐ MRI-based neuroimaging

Antibodies

Antibodies used

Anti-His monoclonal antibody (Santa Cruz Biotechnology Cat# sc-8036, Lot# L0220) using a dilution 1:2000 with an incubation of 2 h; Anti-GST monoclonal antibody (Santa Cruz Biotechnology, Cat# sc-138, sc-138, lot# B2124) using a dilution 1:3000 with an incubation of 2 h.

Validation

Anti-His monoclonal antibody was previously used in Renna. et al (2018 - Nat. Comm.) for the same purpose. Anti-His monoclonal antibody is suitable for WB purpose and it has been widely used with more than 1355 citations (https://www.scbt.com/p/gst-antibody-b-14)

Dual use research of concern

Policy information about dual use research of concern

Hazards

Could the accidental, deliberate or reckless misuse of agents or technologies generated in the work, or the application of information presented in the manuscript, pose a threat to:

No	Yes	
x		Public health
x		National security
x		Crops and/or livestock
x		Ecosystems
x		Any other significant area

Experiments of concern

Does the work involve any of these experiments of concern:

No	Yes	
x		Demonstrate how to render a vaccine ineffective
x		Confer resistance to therapeutically useful antibiotics or antiviral agents
x		Enhance the virulence of a pathogen or render a nonpathogen virulent
x		Increase transmissibility of a pathogen
x		Alter the host range of a pathogen
x		Enable evasion of diagnostic/detection modalities
x		Enable the weaponization of a biological agent or toxin
x		Any other potentially harmful combination of experiments and agents

Plants

Seed stocks	Seeds of the A. thaliana natural accessions (Supplemental Table 1) and the mutant lines ire1a (Col-0; WISCDSLOX420D09), ire1b (Col-0; SAIL_238_F07), bzip28 (Col-0; SALK_132285), bzip60 (Col-0; SALK_050203), bap2 (Col-0; Salk_052789), and bap1 (Col-0; SALK_092421) were obtained from the Arabidopsis Biological Resource Center (Columbus, OH, USA). The bon1 mutant was been previously characterized in Hua et al. (2001 - Genes and Development).
Novel plant genotypes	ire1a ire1b bap2, bzip28 bap2, bzip60 bap2 and bzip18 bzip60 bap2 were generated by crossing bap2 mutant with the corresponding double or single mutants. Experiments were performed in the F3 and F4 generation.<br>The transgenic lines expressing pBAP2Na-1:BAP2, and pBAP2Est:BAP2N67S in bap2 or Est-0 background were generated by Agrobacterium standard floral dip method. Six independent lines for each transformation were analyzed, and two independent lines were selected. Experiments were performed in the T3 and T4 generation.
Authentication	For ire1a ire1b bap2, bzip28 bap2, bzip60 bap2 and bzip18 bzip60 bap2 crosses, T-DNA crosses were analyzed by PCR at F1 and f2 generations.<br>For the transgenic lines, the estimation of the number of loci was performed by analyzing the segregation data of the corresponding antibiotic resistance in the progeny of the different T1 plants. The T2 homozygous plants were identified by segregation analysis of the corresponding antibiotic resistance in the progeny of the selected T2 transgenic plants with a unique insertion. The gene expression was analyzed by RT-qPCR and by fluorescent microscope observation
